# The effect of using acidified turmeric on some productive parameters and intestinal bacterial counts in broilers at high stocking density pens

**DOI:** 10.5455/javar.2022.i572

**Published:** 2022-03-10

**Authors:** Sugiharto Sugiharto, Turrini Yudiarti

**Affiliations:** Faculty of Animal and Agricultural Sciences, Universitas Diponegoro, Semarang, Central Java, Indonesia

**Keywords:** Acidification, broiler, curcumin, overcrowding, stress

## Abstract

**Objective:**

This work investigated the impact of acidified turmeric on growth, blood profile, and gut bacterial counts of broiler chickens stocked in an overcrowding stress condition.

**Materials and Methods:**

A total of 285 14-day-old Lohmann broiler strains were distributed to T0 (chicks receiving basal feed raised at a density of 9 chicks/m^2^), T1 (chicks receiving basal feed raised at 16 chicks/m^2^), T2 (chicks receiving 1% turmeric powder raised at 16 chicks/m^2^), and T3 (chicks receiving 1% acidified turmeric powder raised at 16 chicks/m^2^). Body weight and feed intake were determined weekly. On day 37, blood and intestinal content were collected and analyzed.

**Results:**

Body weight was higher while feed conversion ratio was lower in T0 than in other groups. Compared to T0, T1 had a lower thymus weight. Erythrocytes and hematocrits were greater in T0 than in T2 and T3. Hemoglobin was higher in T0 than in T3. Serum superoxide dismutase differed as T0 < T1 < T2. Ileal coliform was higher in T0 than in T1 and T3. Lactic acid bacteria counts were higher in T0 and T1 than in T2 and T3.

**Conclusions:**

Acidified turmeric was capable of maintaining the relative weight of the immune organ and ameliorating the oxidative stress of the broiler during overcrowding stress.

## Introduction

The demand for broiler meat rises year after year, along with population growth. Farmers must be able to expand their chicken production capacity in order to meet such increased demand [1]. Instead of building new broiler houses, farmers often stock broiler chickens in high-density conditions to maximize the number of chicks per square meter without enlarging the cage area [2]. Aside from the efficiency benefit, stocking chicks at a high density can cause stress, which is detrimental to broiler health and physiological conditions [2,3]. Dietary intervention using synthetic antioxidants has been a promising strategy to augment broiler growth under stressful circumstances [4]. Legislative constraints, however, may make it difficult to use large levels of synthetic antioxidants in stressed broilers as it may have a carcinogenic effect on humans [4]. 

Turmeric powder has long been known to have antioxidant properties, making it useful for ameliorating the detrimental consequences of stress on the health and productivity of broilers [4]. One of the biggest drawbacks of utilizing turmeric powder for broiler supplementation is its limited bioavailability [5]. Acidification has been reported to improve the nutritional and functional properties of plant components. Bayliak et al. [6] documented that acidic treatment enhanced the antioxidant activities of medicinal plants. In the acid solution, curcumin’s solubility and stability improved, which may thereby increase its bioavailability for the host [7]. In this study, turmeric powder was acidified using the fruit filtrate of *Averrhoabilimbi* (*Averrhoa bilimbi* Linn.), which is rich in organic acids, particularly citric acid [8]. 

So far, there has never been any study on the application of acidified turmeric to relieve stress in broilers. The purpose of the present work was to assess the impact of acidified turmeric on productive and health parameters, including growth, blood profile, and gut bacterial counts of broilers housed in overcrowded conditions. 

## Materials and Methods

### Ethical approval

The trial was verified and approved by the Animal Ethics Committee of the Faculty of Animal and Agricultural Sciences, Universitas Diponegoro (No. 57-05/A3/KEP/FPP).

#### Preparation of acidified turmeric

The acidified turmeric was prepared according to the method explained by Sugiharto et al. [9]. The ripe *A. bilimbi* fruit was gathered from the gardens near the faculty. Turmeric was purchased from the traditional market in Semarang, Central Java Province. Acidified turmeric was manufactured by blending turmeric powder with *A. bilimbi* fruit filtrate (1:3; gm:ml). The blend was sun-dried, following incubation at room temperature for 4 days, and then stored in the refrigerator (4°C) until needed. Samples of turmeric powder and acidified turmeric were obtained for analysis. The pH of turmeric powder was 5.90, with 2.34% total acidity, 70.9 ppm antioxidant activity, and 3.47% total phenols. The pH of acidified turmeric was 3.70, with 6.41% total acidity, 81.2 ppm antioxidant activity, and 3.35% total phenols, whereas the total acidity of turmeric powder was 2.34%.

### In vivo experiment

Two hundred eighty-five 14-day-old broiler chicks (Lohmann strains, an average body weight of 370±9.02 gm; mean±standard deviation) were employed in the experiment, which was conducted in accordance with a completely randomized setting. The chicks were reared according to standard raising protocols using prestarter (commercial) feed with 23% crude protein, 5% crude fat, 5% crude fiber, and 7% ash from day 1 until day 14. The birds were randomly distributed into four groups, each with five replicates, starting on day 14. The groups included T0 (chicks stocked at a density of 9 chicks/m^2^ and provided with control/basal feed), T1 (chicks stocked at a density of 16 chicks/m^2^ and provided with basal feed), T2 (chicks stocked at a density of 16 chicks/m^2^ and provided with feed containing 1% turmeric powder), and T3 (chicks stocked at a density of 16 chicks/m^2^ and provided with feed containing 1% acidified turmeric powder). The birds were maintained in an open-sided poultry house with bedding made from rice husks. A hand feeder and a drinker were provided for each pen. Throughout the trial, a continuous illumination regimen was used. The base feed was formulated in accordance with Indonesian National Broiler Feed Standards [10] (Table 1). From day 14 until harvest (day 37), turmeric powder or acidified turmeric powder was applied to feeds (“on top”). The chicks were given the Newcastle disease vaccine (NDV) on day 4 (*via* eye drops) and day 18 (via drinking water). Day 12 also saw the addition of vaccination for infectious bursal disease that was given through the water.

From day 14 to day 37, the chicks’ live body weight, feed consumption, and feed conversion ratio (FCR) were all weekly measured. On day 37, the blood was taken from two male chicks, reflecting the average weight of each cage (10 birds/group), and then slaughtered [9]. The organs of the birds were collected after being de-feathered and dissected. The digesta of the ileum and cecum were collected to count selected bacteria. The contents of the duodenum, jejunum, ileum, and cecum were also obtained for the pH determination (using a digital pH meter; OHAUS ST300). The relative weight of the internal organs was estimated when the internal organs were weighed (in an empty condition).

The blood was divided into two tubes, one with ethylenediaminetetraacetic acid (EDTA) to measure the whole blood counts, and the other with non-EDTA to generate blood serum. The Prima Fully-Auto Hematology Analyzer (PT Prima Alkesindo Nusantara, Jakarta, Indonesia) was used to examine the entire blood profile according to the manufacturer’s guidelines. The blood was permitted to remain at room temperature for 2 h. To create serum, the blood was then centrifuged at 5,000 rpm for 10 min. The antibodies against NDV were measured using the hemagglutination inhibition (HI) technique and presented as a geometric mean (Log2). The levels of superoxide dismutase (SOD) and malondialdehyde (MDA) were determined as described by Agusetyaningsih et al. [11]. The SOD was investigated based on the samples’ ability to reduce pyrogallol autoxidation, while a thiobarbituric acid (TBA) reactive material test was used to determine MDA. 

**Table 1.** Feedstuff and chemical constituents of feed (days 14–37).

Items	(%, unless otherwise noted)
Yellow maize	58.7
Palm oil	2.90
Soybean meal (crude protein of 44.15%)	34.7
DL-methionine, 990 gm	0.19
Bentonite	0.75
Limestone	0.75
Monocalcium phosphate	1.20
Premix^a^	0.34
Chlorine chloride	0.07
Salt	0.40
Chemical constituents:	
ME, (kcal/kg)^b^	3,000
Crude protein	20.0
Crude fiber	5.52
Ca	1.00
P (available)	0.56

a Provides each kg of diet: 1.10 gm Zn, 1.0 gm Mn, 0.85 gm Fe, 75 mg Cu, 4 mg Se, 6 mg Co, 19 mg I, 1.23 gm K, 1.23 gm Mg, 1,250,000 IU vit A, 1.35 gm pantothenic acid, 250,000 IU vit D_3_, 1.88 gm vit E, 250 mg vit K_3_, 250 mg vit B_1_, 750 mg vit B_2_, 500 mg vit B_6_, 2.5 gm vit B_12_, 5.0 gm niacin, 125 gm folic acid, and 2.5 mg biotin.

b ME (metabolizable energy) was determined on the basis of formula: 40.81 {0.87 (crude protein + 2.25 crude fat + nitrogen‐free extract) + 2.5}.

The counts of lactose-negative *Enterobacteriaceae* (LNE) and coliform were counted as colorless and red colonies when grown on MacConkey agar (Merck KGaA, Darmstadt, Germany), following 24 h of incubation (aerobic) at 38°C. *Enterobacteriaceae* includes both coliform and LNE. On De Man, Rogosa and Sharpe agar (Merck KGaA), the numbers of lactic acid bacteria (LAB) were determined following incubation (anaerobic) at 38°C for 48 h [12]. 

### Statistical analysis

An analysis of variance (SPSS 16.0 version) was employed to treat the data. If the treatments showed substantial effects (*p* < 0.05), the Duncan multirange test was further employed.

## Results

### Performance of broiler chickens

Body weight on day 37 and weight gain were greater (*p* < 0.05) in T0 than in T1, T2, and T3. Conversely, FCR was better (*p* < 0.05) in T0 than in T1, T2, and T3 birds. The cumulative feed consumption did not vary (*p* > 0.05) across the groups from days 14 to 37 (Table 2).

### Relative weight of internal organs of broilers

Compared to T0, the birds in T1 showed a lower (*p* < 0.05) weight of thymus, but the variation was not meaningful as compared with T2 and T3 birds (Table 3). The weights of the heart, liver, gizzard, proventriculus, pancreas, gut segments, spleen, and bursa* of Fabricius* across the broiler treatment groups were not different (*p* > 0.05).

### Complete blood profiles of broilers

The levels of erythrocytes and hematocrits were higher (*p* < 0.05) in T0 than in T2 and T3, but were not different from those in T1 broilers (Table 4). The hemoglobin concentration was higher (*p* < 0.05) in T0 than in T3, but was not divergent from that in T1 and T2 broilers. Other parameters were not distinct (*p* > 0.05) among the treatment groups. 

### SOD, malondialdehyde, and antibody titer against NDV

The values of SOD in serum differed (*p* < 0.05) among chickens, as T0<T1<T2<T3 birds (Table 5). The variations in MDA and antibody titers against NDV across broiler chickens were insignificant (*p* > 0.05).

### Intestinal pH and selected bacterial counts of broilers

**Figure 1.**
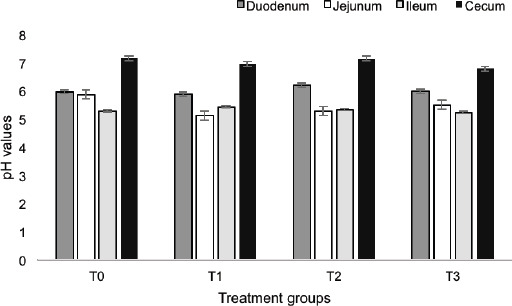
Intestinal pH values of broilers. T0: chicks stocked at a density of 9 chicks/m^2^ and provided with control/basal feed, T1: chicks stocked at a density of 16 chicks/m^2^ and provided with basal feed, T2: chicks stocked at a density of 16 chicks/m^2^ and provided with feed containing 1% turmeric powder, and T3: chicks stocked at a density of 16 chicks/m^2^ and provided with feed containing 1% acidified turmeric powder, SEM: standard error of the mean.

There were no significant differences in the intestinal pH values across broiler chickens (Fig. 1). In the ileum, the counts of coliform were greater (*p* < 0.05) in T0 than in T1 and T3 (Table 6). The counts of LAB were greater (*p* <0.05) in T0 and T1 than in T2 and T3 broilers. There was no meaningful impact (*p* > 0.05) of the intervention on the selected bacteria in the cecum of broilers. 

## Discussion

Data in the current study revealed that housing broilers in high-density situations compromised the growth of chickens. FCR was also significantly aggravated by high-density raising systems, resulting in inefficient feed usage. This was in agreement with Li et al. [13]. Wu et al. [14] showed that stress generated by high density blocked the expression of myogenic genes, hence limiting the muscle development of broilers. In this investigation, dietary turmeric or acidified turmeric powders could not compensate for the stress caused by high stocking density. This differed from the findings of Pimson et al. [15], who found that curcumin derived from *Curcuma longa* (10% of feed) improved the growth of broilers housed at a high density (16 birds/m^2^). The nature and dose of additives administered and environmental conditions throughout the trial, such as temperature, humidity, litter, ammonia condition, etc., all have a role in determining the efficacy of the additive in alleviating the growth-retarded effect of high stocking density. The dose of turmeric and acidified turmeric powder employed in our study was smaller than that used by Pimson et al. [15] and thus did not appear capable of generating a significant impact. Also, they used curcumin (extracted from *C. longa*), which seems to be more powerful in affecting broilers than that of turmeric or acidified turmeric powders. The high environmental temperature (33°C–35°C, at day) and humidity (85–90%, at day) throughout the study appeared to aggravate the stress caused by the high stocking density, and hence turmeric and acidified turmeric powder seemed too hard (ineffective) in compensating the stress on chickens in this present study. Note that temperature and humidity are the two factors that can exacerbate stress due to overcrowding in broilers [16].

**Table 2.** Production parameters of broilers (days 14–37).

Items	T0	T1	T2	T3	SEM	*p*-value
Final body weight (gm)	1826^a^	1561^b^	1549^b^	1575^b^	28.9	<0.01
Body weight gain (gm)	1448^a^	1190^b^	1188^b^	1206^b^	28.1	<0.01
Cumulative feed intake (gm)	2639	2629	2648	2638	12.8	0.97
FCR	1.82^b^	2.21^a^	2.23^a^	2.19^a^	0.04	<0.01

^a,b^Means in the corresponding row with superscript letters are different (*p* < 0.05).

T0: chicks stocked at a density of 9 chicks/m^2^ and provided with control/basal feed, T1: chicks stocked at a density of 16 chicks/m^2^ and provided with basal feed, T2: chicks stocked at a density of 16 chicks/m^2^ and provided with feed containing 1% turmeric powder, and T3: chicks stocked at a density of 16 chicks/m^2^ and provided with feed containing 1% acidified turmeric powder; FCR: feed conversion ratio, SEM: standard error of the means.

**Table 3.** Relative weight of internal organs of broilers.

Items (% live BW)	T0	T1	T2	T3	SEM	p-value
Heart	0.42	0.44	0.42	0.39	0.01	0.64
Liver	2.55	2.67	2.54	2.39	0.10	0.83
Proventriculus	0.49	0.56	0.52	0.49	0.02	0.69
Gizzard	1.57	1.61	1.54	1.65	0.05	0.87
Pancreas	0.24	0.28	0.23	0.30	0.01	0.13
Duodenum	1.03	1.00	1.02	0.84	0.06	0.68
Jejunum	1.45	1.31	1.07	1.24	0.06	0.11
Ileum	0.71	0.81	0.63	0.86	0.05	0.42
Caeca	0.68	0.60	0.55	0.60	0.02	0.30
Spleen	0.12	0.13	0.10	0.06	0.01	0.37
Thymus	0.22^a^	0.08^b^	0.16^ab^	0.15^ab^	0.02	0.04
Bursa of Fabricius	0.09	0.08	0.09	0.08	0.01	0.97

^a,b^Means in the corresponding row with superscript letters are different (*p* < 0.05).

T0: chicks stocked at a density of 9 chicks/m^2^ and provided with control/basal feed, T1: chicks stocked at a density of 16 chicks/m^2^ and provided with basal feed, T2: chicks stocked at a density of 16 chicks/m^2^ and provided with feed containing 1% turmeric powder, and T3: chicks stocked at a density of 16 chicks/m^2^ and provided with feed containing 1% acidified turmeric powder, BW: body weight, SEM: standard error of the mean.

**Table 4.** Blood cell counts of broiler chickens.

Items	T0	T1	T2	T3	SEM	*p*-value
Erythrocytes (10^12^/l)	4.51^a^	3.84^ab^	3.11^b^	2.73^b^	0.21	<0.01
Hemoglobin (gm/dl)	14.2^a^	12.3^ab^	11.4^ab^	9.70^b^	0.53	0.02
Hematocrits (%)	56.9^a^	47.9^ab^	36.7^b^	34.1^b^	2.73	<0.01
MCV (fl)	127	125	126	126	0.47	0.77
MCH (pg)	32.0	34.3	35.3	36.4	0.61	0.06
MCHC (gm/dl)	25.4	27.3	29.0	28.2	0.49	0.05
RDW-SD (10^−15^ l)	48.3	48.6	54.3	52.8	1.19	0.19
RDW-CV (%)	10.1	10.2	11.3	11.1	0.24	0.16
MPV (10^−15^ l)	7.46	7.98	7.42	7.42	7.47	0.88
PDW (%)	11.0	13.3	10.7	12.0	0.73	0.60
Leukocytes (10^9^/l)	126	102	99.8	90.9	6.61	0.27
Heterophils (10^9^/l)	7.15	11.4	8.80	9.35	0.93	0.46
Lymphocytes (10^9^/l)	119	90.2	91.0	81.5	5.95	0.12
Thrombocytes (10^9^/l)	127	88.7	43.9	51.7	19.6	0.43

^a,b^Means in the corresponding row with superscript letters are different (*p* < 0.05).

T0: chicks stocked at a density of 9 chicks/m^2^ and provided with control/basal feed, T1: chicks stocked at a density of 16 chicks/m^2^ and provided with basal feed, T2: chicks stocked at a density of 16 chicks/m^2^ and provided with feed containing 1% turmeric powder, and T3: chicks stocked at a density of 16 chicks/m^2^ and provided with feed containing 1% acidified turmeric powder; MCH: mean corpuscular hemoglobin, MCV: mean corpuscular volume, MCHC: mean corpuscular hemoglobin concentration, RDW-CV: red blood cell distribution width-coefficient variation, RDW-SD: red blood cell distribution width-standard deviation, PDW: platelet distribution width, MPV: mean platelet volume, SEM: standard error of the mean.

**Table 5.** SOD, MDA, and antibody titer against NDV.

Items	T0	T1	T2	T3	SEM	*p*-value
SOD (U/ml)	35.6^d^	41.7^c^	43.7^b^	47.9^a^	0.77	<0.01
MDA (nmol/ml )	2.41	2.75	2.49	2.23	0.09	0.19
Antibody titers toward NDV (Log_2_ GMT)	5.20	5.40	6.00	5.00	0.27	0.61

^a,b,c^Means in the corresponding row with superscript letters are obviously different (*p* < 0.05).

T0: chicks stocked at a density of 9 chicks/m^2^ and provided with control/basal feed, T1: chicks stocked at a density of 16 chicks/m^2^ and provided with basal feed, T2: chicks stocked at a density of 16 chicks/m^2^ and provided with feed containing 1% turmeric powder, and T3: chicks stocked at a density of 16 chicks/m^2^ and provided with feed containing 1% acidified turmeric powder; MDA: malondialdehyde, SOD: superoxide dismutase, GMT: geometric mean titers, NDV: Newcastle disease, SEM: standard error of the mean.

**Table 6.** Selected bacterial counts of the intestine of broilers.

Items	T0	T1	T2	T3	SEM	*p*-value
Ileum						
Coliform (log CFU/gm)	6.40^a^	5.26^b^	5.61^ab^	5.26^b^	0.17	0.04
LNE (log CFU/gm)	5.96	5.26	5.79	5.26	0.17	0.37
Enterobacteriaceae (log CFU/gm)	6.47	5.26	5.80	5.26	0.20	0.10
LAB (log CFU/gm)	10.8^a^	9.86^a^	7.90^b^	7.88^b^	0.35	<0.01
LAB/coliform ratio	1.72	1.88	1.43	1.50	0.07	0.06
Cecum						
Coliform (log CFU/gm)	5.78	5.76	5.26	6.51	0.26	0.44
LNE (log CFU/gm)	5.26	6.62	5.26	6.30	0.25	0.10
Enterobacteriaceae (log CFU/gm)	5.82	6.66	5.26	7.09	0.31	0.14
LAB (log CFU/gm)	10.5	10.8	10.3	10.7	0.15	0.59
LAB/coliform ratio	1.86	1.92	1.95	1.73	0.07	0.68

^a,b^Means in the corresponding row with superscript letters are different (*p* < 0.05).

T0: chicks stocked at a density of 9 chicks/m^2^ and provided with control/basal feed, T1: chicks stocked at a density of 16 chicks/m^2^ and provided with basal feed, T2: chicks stocked at a density of 16 chicks/m^2^ and provided with feed containing 1% turmeric powder, and T3: chicks stocked at a density of 16 chicks/m^2^ and provided with feed containing 1% acidified turmeric powder; LAB: lactic acid bacteria, LNE: lactose-negative Enterobacteriaceae, CFU: colony-forming unit, SEM: standard error of the mean.

When compared to broilers kept at a normal density, the weight of the thymus was less in broilers housed in overcrowded pens. This was in line with the findings of Mahmoud and El-Rayes [3], who confirmed that increased density reduced the relative weight of broiler thymus. Likewise, Li et al. [2] documented that stress caused by high-density conditions was linked to a decrease in the bursaof Fabricius weight in broilers. It seems that overcrowding stress induces oxidative stress, which may, in turn, impair the development of immune organs in chickens [3]. Interestingly, the dietary incorporation of turmeric or acidified turmeric powder was capable of maintaining the relative weight of the thymus during the overcrowding stress. In this case, the antioxidative properties of curcumin in turmeric or acidified turmeric seemed to be responsible for counteracting the harmful impact of oxidative stress on broiler lymphoid organs [4]. 

In this investigation, the values of red blood cells and hematocrits were found to be lower in high-density stocked birds receiving turmeric or acidified turmeric powder than in normal-density stocked birds. A previous study reported that overcrowding increased red blood cell values in broilers [17]. Owing to the role of red blood cells in transporting oxygen, the elevated red blood cells were assumed to be due to an increased oxygen demand for metabolic activities in broilers in response to stress [16]. However, this did not appear to be the case in our investigation, as no significant variations in erythrocytes, hemoglobin, or hematocrits were seen between high- and normal-density stocked birds receiving the basal feed. Turmeric has been shown to reduce erythrocyte count, hematocrits, hemoglobin concentration, and mean corpuscular hemoglobin concentration (MCHC) in rabbits [18]. According to Luber et al. [19], turmeric supplementation can cause liver damage. Because the liver plays such an important part in erythropoiesis, liver damage may result in fewer erythrocytes being formed. The latter condition was, therefore, associated with the lower hemoglobin concentration and hematocrit values of broilers. In this regard, long-term use of turmeric may be detrimental to the liver’s health and functions. 

Stress is usually related to elevated SOD and MDA values, which is a defensive reaction of chicks toward surplus free radicals [4]. However, depending on the severity of the stress, the changes in SOD and MDA levels are not always consistent for overcrowding-induced stress. According to Pimson et al. [15], high stocking density increased broiler SOD levels. On the contrary, Li et al. [2] discovered that stress caused by high stocking density decreased broiler SOD levels. The increased SOD levels in broilers housed in high-density pens appeared to represent a response of broilers to excessive free radical production in this investigation, which is consistent with Pimson et al. [15] and Sugiharto et al. [20]. In support of this inference, Magnuson et al. [21] also revealed that high stocking density increased glutathione (potent nonenzymatic antioxidant) concentration in the plasma of chickens. Irrespective of the high-density influence, dietary administration of turmeric or acidified turmeric powders increased SOD levels of broilers during this investigation. This finding was in agreement with Pimson et al. [15], who confirmed that dietary curcumin increased SOD levels in broilers. Also, Zhang et al. [22] also reported that curcumin derived from turmeric increased the antioxidant activity (SOD activity) of broilers. Based on the above studies, we inferred that dietary administration of turmeric or acidified turmeric powders could increase SOD levels of broilers in this study. The fact that MDA levels were reduced (although not statistically significant) when turmeric or acidified turmeric powder was administered (as compared with that in broilers housed at a high stocking density and receiving only basal feed), further supporting our inference that turmeric or acidified turmeric powder improves the antioxidant status of broilers. This study discovered that high-stocked broilers that received acidified turmeric had higher SOD levels and lower MDA levels than those that received turmeric powder. This may suggest that acidification could improve the application of turmeric as an antioxidant source for broilers. Note that, as previously mentioned, the acidification process increased the antioxidant activity of turmeric by 12.7%. There was no way to measure the bioavailability of the materials in this study because we did not conduct *in vitro* digestion [23] to see how they were broken down. 

Stocking broilers in high-density pens has been observed to increase coliform populations in the intestines of chicks [24]. On the contrary, the current study found that raising broilers in a high-density pen lowered coliform bacteria counts. The exact cause of this condition was unknown, but it appeared that lower counts of coliform in the intestine of broilers housed at a high stocking density were caused by the decreased nutrient availability (serving as a substrate for bacteria in the intestine) due to lower digestibility of feed in stressed broilers [4]. In terms of LAB counts, our data indicated that stocking broilers in high-density pens did not affect the gut LAB counts. On the contrary, Cengiz et al. [25] found that high-density conditions decreased the number of Lactobacilli in broiler intestines. The severity of stress (due to stocking density), broiler house hygiene, feed composition, and other factors appeared to determine the extent of the adverse effect of high density on bacterial composition in broiler intestines. According to the literature, LAB is curcumin sensitive [26]. The antibacterial activity of the curcumin content in turmeric may, therefore, be attributed to the lower LAB counts in the ileum of broilers in the current investigation. However, this inference should be viewed with caution as Namagirilakshmi et al. [27] found that incorporation of turmeric meal enhanced *Lactobacillus* numbers in the intestines of broilers. 

Apart from the results presented and discussed above, there are some limitations to this current study, which may require caution in interpreting the data obtained. The limited number of replicates for evaluating performance data (five pens for each treatment group) may lead to bias due to the high potential for error. On the other hand, the potential for error may be smaller in parameters other than performance because the number of replications for each group is considered sufficient (10 samples for each treatment group). Another limitation is the absence of data on the bioavailability of turmeric and acidified turmeric because we did not perform an in vitro digestion test to measure the bioavailability of these two herbal ingredients. The absence of such bioavailability data makes it difficult to compare the effects of turmeric and acidified turmeric, especially with regard to the SOD parameters, where there was a significant difference between chickens receiving turmeric and acidified turmeric.

## Conclusion

High stocking density impaired performance, compromised immune organ development, and induced oxidative stress. The use of acidified turmeric was able to maintain the development of the thymus and ameliorate oxidative stress during the overcrowding stress.

## List of abbreviations

LAB: Lactic acid bacteria, SOD: Superoxide dismutase, MDA: Malondialdehyde, FCR: Feed conversion ratio, EDTA: Ethylenediaminetetraacetic acid, NDV: Newcastle disease vaccine, HI: Hemagglutination inhibition, TBA: Thiobarbituric acid, MCHC: Mean corpuscular hemoglobin concentration
